# Evaluation of a standardized instrument for post hoc analysis of trauma-team-activation-criteria in 75,613 injured patients an analysis of the TraumaRegister DGU^®^

**DOI:** 10.1007/s00068-021-01668-2

**Published:** 2021-04-19

**Authors:** Dan Bieler, Heiko Trentzsch, Axel Franke, Markus Baacke, Rolf Lefering, Thomas Paffrath, Lars Becker, Helena Düsing, Björn Heindl, Kai Oliver Jensen, Orkun Oezkurtul, Uwe Schweigkofler, Kai Sprengel, Bernd Wohlrath, Christian Waydhas, the Committee on Emergency Medicine, Intensive Care and Trauma Management (Sektion NIS) of the German Trauma Society (DGU)

**Affiliations:** 1 Department of Orthopaedics and Trauma Surgery, Heinrich Heine University Hospital Düsseldorf, Moorenstraße 5, 40225 Düsseldorf, Germany; 2 Department of Trauma Surgery and Orthopaedics, Reconstructive and Hand Surgery and Burn Medicine, German Armed Forces Central Hospital Koblenz, Koblenz, Germany; 3 Institut Für Notfallmedizin Und Medizinmanagement (INM), Klinikum der Universität München LMU München, Munich, Germany; 4 Department of Trauma and Reconstructive Surgery/Emergency Department, The Krankenhaus der Barmherzigen Brüder Trier (a Brothers of Mercy hospital), Trier, Germany; 5 Faculty of Health, Witten/Herdecke Private University, Institute for Research in Operative Medicine (IFOM), Cologne, Germany; 6 Department of Trauma Surgery, Hospital of the Augustinians “Severinsklösterchen”, Cologne, Germany; 7 Department of Trauma Surgery, Hand and Reconstructive Surgery, University Hospital Essen, Essen, Germany; 8 Department of Trauma-, Hand- and Reconstructive Surgery, University Hospital Muenster, Muenster, Germany; 9 Department of Trauma and Reconstructive Surgery, Diakonie Clinic Jung Stilling Siegen, Siegen, Germany; 10 Department of Trauma, University Hospital Zurich (USZ), University of Zurich (UZH), Zurich, Switzerland; 11 Department of Surgical Medicine, Surgery I, University Hospital Leipzig, Leipzig, Germany; 12 Department of Orthopaedic and Trauma Surgery, Berufsgenossenschaftliche Unfallklinik Frankfurt, Frankfurt am Main, Germany; 13 Department of Surgery, BG University Hospital Bergmannsheil, Bochum, Germany; 14 Medical Faculty of the University of Duisburg-Essen, Essen, Germany

**Keywords:** Trauma-team-activation, Field triage, TraumaRegister DGU^®^, Trauma care, Severe injury

## Abstract

**Introduction:**

To improve the quality of criteria for trauma-team-activation it is necessary to identify patients who benefited from the treatment by a trauma team. Therefore, we evaluated a post hoc criteria catalogue for trauma-team-activation which was developed in a consensus process by an expert group and published recently.

The objective was to examine whether the catalogue can identify patients that died after admission to the hospital and therefore can benefit from a specialized trauma team mostly.

**Materials and methods:**

The catalogue was applied to the data of 75,613 patients from the TraumaRegister DGU^®^ between the 01/2007 and 12/2016 with a maximum abbreviated injury score (AIS) severity ≥ 2. The endpoint was hospital mortality, which was defined as death before discharge from acute care.

**Results:**

The TraumaRegister DGU^®^ dataset contains 18 of the 20 proposed criteria within the catalogue which identified 99.6% of the patients who were admitted to the trauma room following an accident and who died during their hospital stay. Moreover, our analysis showed that at least one criterion was fulfilled in 59,785 cases (79.1%). The average ISS in this group was 21.2 points (SD 9.9). None of the examined criteria applied to 15,828 cases (average ISS 8.6; SD 5). The number of consensus-based criteria correlated with the severity of injury and mortality. Of all deceased patients (8,451), only 31 (0.37%) could not be identified on the basis of the 18 examined criteria. Where only one criterion was fulfilled, mortality was 1.7%; with 2 or more criteria, mortality was at least 4.6%.

**Discussion:**

The consensus-based criteria identified nearly all patients who died as a result of their injuries. If only one criterion was fulfilled, mortality was relatively low. However, it increased to almost 5% if two criteria were fulfilled. Further studies are necessary to analyse and examine the relative weighting of the various criteria.

**Summary:**

Our instrument is capable to identify severely injured patients with increased in-hospital mortality and injury severity. However, a minimum of two criteria needs to be fulfilled. Based on these findings, we conclude that the criteria list is useful for post hoc analysis of the quality of field triage in patients with severe injury.

## Introduction

Severe trauma is one of the most frequent causes of death in patients under 45 years of age and is primarily caused by traffic accidents and falls from heights [1–3]. The management of these patients constitutes an enormous medical, logistic, and socio-economic challenge due to the complexity of injuries, medical support around the clock, and the necessity of rapid and careful action in the shortest time possible and involving various medical fields [4, 5]. Today it is generally agreed that trauma room management and initial care are of prime importance for the survival of patients.

A series of preclinical situations and conditions (field triage criteria) have been established. Should they occur, the trauma room should be notified and, as a rule, the trauma team should be activated (Level 3 guideline on the treatment of patients with severe/multiple injuries, American College of Surgeon (ACS) criteria, Guidelines for Field Triage of Injured Patients by CDC) [6–8]. These criteria include the disruption of vital functions, obvious severe injuries, and accident mechanisms. Trauma team activation criteria are often based on a certain injury severity (e.g. an Injury Severity Score (ISS) of 16 points or more [9, 10]), death in the emergency department, admission to an intensive care unit, or the necessity of life-saving surgery or interventions [11]. While there is little data on the extent of over- and undertriage in Germany, figures published in other countries differ considerably. For example, overtriage rates vary between 12 and 85% and undertriage rates between 0.4% and 21%. Publications from the United States show that, despite an overtriage rate of 72%, undertriage rates are still between 10 and 19% [7, 12–14]. Studies from France, whose emergency medical system is more similar to the German system than that of North America, present a different picture. These studies report an overtriage rate of 60% and an undertriage rate of merely 1% [15, 16]. The considerable differences noted here depend not least on the different criteria used to define overtriage and undertriage.

The criteria on trauma team activation in the German Level 3 guideline have been in the focus of an intense debate for a number of years. This debate revolves around the predictive value of the field triage criteria; in particular, whether B criteria (trauma team activation on account of the type of accident) unnecessarily increase the number of patients who, from a medical point of view, do not require trauma room care with full trauma team activation. Patients who are admitted via trauma room with full trauma team activation and who do not require this level of care even though they do not need it consume unnecessarily valuable resources (over-triage). Patients who would have required trauma team activation but who bypass the trauma room because they were missed by field triage criteria and thus did not receive appropriate care are rated as under-triaged. While over-triage places a strain on resources and thus involves economic and procedural risks, under-triage involves the risk that patients receive insufficient care and may, in extreme cases, even suffer unfavourable outcome. There are practically no studies that examine the quality of triage decisions in Germany based on the Level 3 guideline.

Thus, little is known on the true rate of over and under-triage and weather resources are used optimally. The reason why such studies are difficult to conduct is that there was no commonly accepted golden standard for deciding whether a patient has benefited from trauma room care or not. Such retrospective classification is necessary to distinguish between true positive, true negative, false-positive and false-negative cases. This, however, is the basic requirement to be able to estimate over-triage and under-triage meaningfully.

Recently, the Committee on Emergency Medicine, Intensive Care and Trauma Management (Sektion NIS) of the German Trauma Society (DGU) prepared a consensus-based criteria catalogue (see Table 1) that serves as a standardised instrument for classifying severely injured patients post hoc with regard to the quality of triage [15]. According to this consensus, treatment in the resuscitation bay by a trauma team is necessary when one of these criteria is fulfilled. If it was provided, triage is true positive.

**Table 1:** Consensus-based criteria catalogue for the retrospective identification of patients requiring trauma room care [17]

Injury severity
Abbreviated injury scale (AIS) severity ≥ 4^TR^
Intensive medical care (without intermediate care)
ICU stay > 24 h^TR^
Mortality
Death within 24 h^TR^
Invasive measures (prehospital or in trauma room)^&^
Resuscitation^TR^
Advanced airway management^TR^ Chest tube or needle decompression
Pericardiocentesis
Application of tourniquet (prehospital)
Administration of catecholamines^TR^
Transfusion^TR^ Chest tube^TR^
Surgical/radiological therapeutic intervention*
Life-saving/organ-saving^TR^ extremity-saving surgery^#^
Radiological therapeutic intervention^§, TR^
≥ 2 external fixators (humerus, femur, pelvis)^TR^
Impaired vital functions
Pulse oximetry (SpO_2_) < 90% ^TR^
Respiratory rate < 9 or > 29/min ^TR^ Systolic blood pressure < 90 mmHg
Shock index > 0.9^TR^
Systolic blood pressure < 90 mmHg^TR^
Glasgow coma scale (GCS) < 9^TR^
Drop in GCS of 2 points or more prior to admission^TR^
Hypothermia < 35^TR^

If at least one criterion is fulfilled, trauma room care provided by a trauma team is considered necessary

^**&**^Not including intraoperative invasive measures or measures to prepare for non-emergency surgery (e.g. intubation)

*Performed in the emergency department or immediately after, but prior to admission to intensive care (or another department)

^§^Only therapeutic measures such as embolisation, coiling, and stenting

^TR^Verifiable and verified on the basis of TraumaRegister DGU^®^

To verify whether the catalogue can correctly identify the need for trauma team activation, we carried out a validation process on the basis of TraumaRegister DGU^®^ data. The goal was to examine whether the catalogue can identify severely injured patients with an increased mortality risk to evaluate in the future especially with regard to the positive predictive value of new and existing activation criteria for trauma teams.

## Materials and methods

TraumaRegister DGU^®^ of the German Trauma Society (Deutsche Gesellschaft für Unfallchirurgie, DGU) was founded in 1993. The purpose of this multi-centre database is to collect pseudonymised data on severely injured patients in a standardised manner.

Data are collected prospectively in four consecutive phases: (A) prehospital phase, (B) trauma room and subsequent surgery, (C) intensive care, and (D) discharge. Data include detailed information on demographics, injury patterns, comorbidities, prehospital and clinical management, intensive care, important laboratory findings including data on transfusion, and outcome. The inclusion criterion is admission to the hospital via the trauma room followed by intensive care or arrival at the hospital with vital signs and death before transfer to intensive care.

The infrastructure for documentation, data management, and data analysis is provided by the Academy for Trauma Surgery (Akademie der Unfallchirurgie GmbH), which is affiliated with the German Trauma Society. Scientific supervision is provided by the Committee on Emergency Medicine, Intensive Care and Trauma Management (Sektion NIS) of the German Trauma Society. Participating hospitals submit pseudonymised data to a central database via a web-based application. Scientific studies are authorised in accordance with a peer-review process, which is stipulated in the publication guideline of the German Trauma Society.

Participating hospitals are primarily located in Germany (90%), but an increasing number of hospitals from other countries contribute data as well (Austria, Belgium, China, Finland, Luxembourg, Slovenia, Switzerland, the Netherlands, and the United Arab Emirates). Currently, approximately 33,000 cases from more than 650 hospitals are entered into the database every year. Participation in TraumaRegister DGU^®^ is voluntary. Hospitals in TraumaNetzwerk DGU^®^, however, are required to enter at least a basic set of data for reasons of quality assurance.

We included data from adult patients (age ≥ 16) treated in Germany and documented with the standard dataset between the years 2007 and 2016. We excluded patients with a maximum injury severity of 1 according to the abbreviated injury scale (AIS). Patients transferred in as well as patients transferred out within 48 h were excluded since admission data or final outcome were missing, respectively.

Statistical analysis was carried out using SPSS (Version 23, IBM Inc., Armonk, NY, USA). Number of cases with percentage or mean with standard deviation (SD) were used for descriptive analysis of categorical and metric variables, respectively. Missing values were excluded on a case-by-case basis.

This study has been performed in accordance with the ethical standards laid down in the 1964 Declaration of Helsinki and its later amendments. It was performed in accordance with the publication guideline of TraumaRegister DGU^®^ and is registered as TR-DGU Project ID 2017–024. According to the guidelines of the responsible state medical association, an ethical vote was not necessary for retrospective anonymous analysis.

## Results

We were able to examine 18 of 20 criteria of the consensus-based catalogue using TraumaRegister DGU^®^ data.

Our analysis showed that 75,613 TraumaRegister DGU^®^ patients who were evaluated, 59,785 cases (79.1%) fulfilled at least one criterion. The average ISS of this group was 21.2 points (SD 9.9). In 15,828 cases, none of the 18 evaluated criteria applied (average ISS 8.6; SD 5.0).

**Fig. 1:**
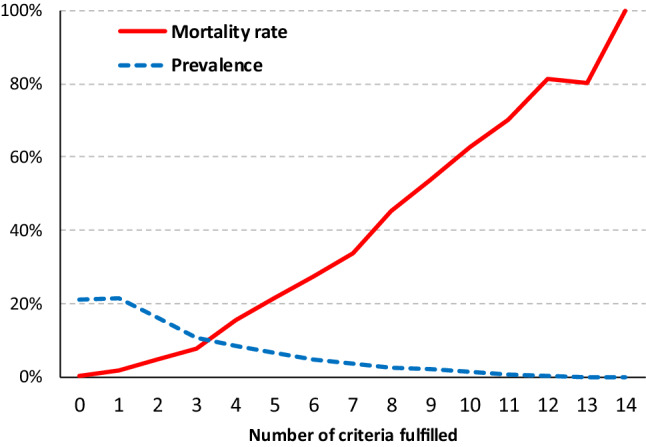
Criteria prevalence and Mortality in relation to criteria prevalence

Table 2 provides an overview of the prevalence of each criterion and the related mortality rate. Depending on the criterion, mortality varied between 9.3% (intensive care >  = 2 calendar days) and 76.2% (CPR). It was evident that higher mortality rates occurred when several criteria were fulfilled at the same time. (Table 3, Fig. 1). Only one criterion applied in 16,365 cases; in almost two-thirds of all cases, this criterion was the duration of ICU stay. In the group with only one criterion fulfilled, the highest mortality rate was 2.3% and thus comparatively low. When none of the catalogue criteria were fulfilled, mortality was only 0.2% (*n* = 31).

**Table 2:** Prevalence of criteria and mortality

Criterion	Prevalence		Mortality		Prevalence, only this criterion		Mortality, only this criterion	
	*N*	%	*N*	%	*n*	%	*n*	%
AIS ≥ 4	28,798	38.1	7162	24.9	1551	5.4	35	2.3
Intensive care ≥ 2 calendar days	46,208	61.1	4308	9.3	10,545	22.8	201	1.9
Died within 24 h	4122	5.5	4122	100.0	26	0.6	26	100.0
Cardio-pulmonary resuscitation (CPR)	3162	4.2	2409	76.2	14	0.4	0	0.0
Advanced Airway	22,771	30.1	6154	27.0	592	2.6	3	0.5
Chest tube	8823	11.7	2033	23.0	263	3.0	0	0.0
Administration of catecholamine	13,150	17.4	4692	35.7	94	0.7	0	0.0
Blood transfusion	7712	10.2	2439	31.6	66	0.9	0	0.0
GCS score < 9	15,099	20	5660	37.5	166	1.1	0	0.0
Drop in GCS ≥ 2	3706	4.9	477	12.9	420	11.3	6	1.4
Systolic blood pressure < 90 mmHg	11,212	14.8	3322	29.6	186	1.7	0	0.0
SpO_2_ < 90%	9484	12.5	2989	31.5	514	5.4	7	1.4
Hypothermia < 35 °C	3040	4	880	28.9	88	2.9	1	1.1
Shock index > 0.9	17,720	23.4	3165	17.9	1639	9.2	3	0.2
Respiratory rate < 9 or > 29	3207	4.2	1452	45.3	45	1.4	1	2.2
Life-saving surgery	6030	8	1642	27.2	126	2.1	0	0.0
Radiological therapeutic intervention	419	0.6	73	17.4	19	4.5	0	0.0
2 or more external fixators (humerus, femur, tibia, pelvis)	937	1.2	118	12.6	11	1.2	0	0.0

*AIS* abbreviated injury scale, *GCS* glasgow coma scale, *SpO*_*2*_ saturation of peripheral oxygen

**Table 3:** Mortality in relation to the number of criteria fulfilled

Number of fulfilled criteria	*n*	Died		Injury severity score	
				Average	SD
0	15,828	31	0.2%	8.6	5.0
1	16,365	283	1.7%	12.1	6.5
2	12,287	562	4.6%	17.8	9.2
3	8134	616	7.6%	19.9	9.7
4	6376	1003	15.7%	23.6	10.6
5	4922	1060	21.5%	26.4	11.3
6	3609	993	27.5%	29.4	13.0
7	2687	910	33.9%	32.6	14.0
8	2026	915	45.2%	35.5	14.8
9	1517	814	53.7%	40.0	15.9
10	1011	634	62.7%	44.0	16.3
11	562	395	70.3%	47.2	16.1
12	233	189	81.1%	47.7	15.0
13	51	41	80.4%	49.9	15.3
14	5	5	100.0%	45.8	12.0
Total	75,613	8451	11.2%	18.6	13.1

*SD* standard deviation

These 31 cases constitute 0.37% of all 8451 deaths. Table 3 shows all patients without any consensus-based risk criteria who died. It should be noted that, in this subgroup, the average age of 75.7 years is much higher than the average age of the overall group (48.1 years), death occurred at the earliest on the third day of the hospital stay (minimum 3 days, maximum 73 days), and the average ISS of 10.7 was far below the overall group (18.6). Further, we observed that most of these patients were not treated on the intensive care unit; 15 of these patients did not receive any intensive care at any time.

## Discussion

The objective of our study was to examine the recently published consensus-based criteria [17] for the activation of a trauma team on the basis of TraumaRegister DGU^®^ data. Almost all of the criteria could be evaluated by the data of the registry. We were unable to verify the criteria “application of a tourniquet” and “performance of pericardiocentesis” using TraumaRegister DGU^®^ as it does not yet include data on these criteria. According to the literature, the frequency of cardiac tamponade is 0.04% for blunt trauma and as high as 6% for penetrating trauma [18–20]. Penetrating injuries are present in approximately only 4% of all severely injured patients in Germany. For this reason, it is rarely necessary to perform pericardiocentesis in trauma patients [21]. The prehospital application of tourniquets has been on the rise only since late 2016. As a result, the significance of this variable can only be evaluated in the future.

We found that consensus-based criteria covered nearly all patients who died. For this reason, the chances of incorrectly assessing a patient are negligible with these criteria with regard to mortality.

In our study group of more than 75,000 patients, we also found that accident-related mortality and severity of injury increase with the number of applicable criteria. That shows that relevant criteria were chosen in the consensus-based process. It is important to note that a single criterion often cannot reflect the complexity of severely injured patients.[10] When only one criterion was present, mortality was at most 2.3% (AIS ≥ 4). The mortality was 0% when the only criterion was a respiration rate of < 9 or > 29 breaths per minute, an ICU stay > 2 days, a drop in GCS of ≥ 2 points, SpO_2_ < 90%, hypothermia < 35, advanced airway and a shock index > 0.9. The criterion “died within 24 h” deserves a special mention in this context. Of course, if it is present, the death rate is 100%. However, the criterion “died within 24 h” is only in 0.6% (*n* = 26) of the cases as a single criterion present. Furthermore, it is particular with a prevalence of only 5.5% a rather rare criterion compared to the other criteria.

It should be noted that possible criteria for trauma team activation, which are yet to be defined, should take various aspects into consideration. Table 2 indicates that perhaps not all post-hoc criteria are highly relevant, and it may be possible to reduce the post-hoc criteria catalogue. In addition, tourniquet and pericardiocentesis could not be evaluated, although it should be noted that these criteria would most likely be coincident with the evaluated criteria. The advantage of these two criteria is that they could also be assessed in the prehospital setting and therefore could be good trauma team activation criteria.

Mortality as an outcome parameter is defined clearly and well documented [22]. To evaluate the quality of trauma-treatment, more aspects like functional results or quality of life might be important parameters for further studies.

In many cases, initial treatment already is indicatory for a good functional outcome [5]. One example is a spinal injury with neurological symptoms. Although the functional outcome is not taken into consideration, the authors nevertheless believe that mortality is a suitable outcome parameter for activation criteria because trauma teams are primarily activated for the treatment of life-threatening injuries. From this perspective, the identification of 99.6% of cases by means of consensus-based criteria is sufficient. This rate is higher than some described in the current literature [7, 23] and is comparable to figures published by other author groups [24].

The fact that 31 deceased patients did not fulfil any consensus-based risk criteria should not be considered to be a fault of the criteria. Whether these deceased patients (Table 4) would have been detected by the two non-verifiable criteria is highly unlikely as injuries requiring pericardiocentesis or a tourniquet generally coincide with a much higher ISS and severe disturbance of vital functions [25]. In view of the advanced age of most of these patients, it is possible that an advance health care directive, a living will or patient wish communicated by family members prevented further treatment. A number of lethal courses (without any of the consensus-based criteria) could have been caused by complications that were not connected to the activation of a trauma room team, for example, thromboembolic events (*n* = 5) and multi-organ failure (*n* = 7). This argument is supported by the fact that the earliest death was observed on the third day of hospital stay (minimum 3 days, maximum 73 days).

**Table 4:** Deceased patients who did not fulfil a criterion

No	Age	Sex	Max. AIS	ISS	ICU stay (d)	Hospital stay (d)	Sepsis	Multiple organ failure	Thromboembolic event
1	19	M	3	9	0	73	No data	No data	No data
2	37	M	3	10	0	7	No	Yes	No data
3	52	M	3	9	1	7	No	No	Yes
4	64	M	2	5	0	3	No data	No data	No data
5	64	F	3	10	0	3	No	Yes	No
6	67	F	3	17	1	7	No	No	No data
7	72	M	3	27	1	12	No	No	Yes
8	73	M	3	13	0	9	No	No	No
9	73	M	3	17	0	9	No	No	No
10	75	M	2	8	0	8	No data	No data	No
11	76	M	3	22	1	33	Yes	No	No
12	77	M	3	13	1	3	No	No	No data
13	77	M	3	13	1	3	No	No	No
14	78	M	3	19	1	5	No	No	No
15	80	M	2	6	0	13	No data	No data	No data
16	80	M	2	12	0	40	No	No	Yes
17	80	M	2	4	0	33	No	No	Yes
18	80	F	3	10	0	62	No data	No data	No
19	83	M	2	8	1	3	Yes	Yes	No
20	83	F	3	9	0	8	No data	No data	No
21	83	F	2	9	1	3	Yes	Yes	No
22	84	M	2	5	1	5	Yes	Yes	No data
23	85	M	3	9	1	3	No	No	No data
24	86	M	3	9	1	6	No	Yes	No
25	86	F	3	10	1	5	No	Yes	No
26	86	M	3	11	1	5	No	No	No
27	88	M	2	5	0	8	No	No	No
28	88	M	3	9	0	21	No	No	No
29	89	M	3	9	0	3	No data	No data	No
30	89	M	3	9	1	4	No	No	No
31	92	F	2	5	1	3	No	No	Yes
	75.7 years	M = 77%	Average 2.7	Average 10.7	0.5 d	13.1 d	Yes = 4	Yes = 7	Yes = 5

*AIS* abbreviated injury scale, *ISS* injury severity score, *ICU* intensive care unit

It should be emphasized that some criteria (e.g. duration of intensive care treatment) can only be assessed post hoc, but in view of our findings, it should be considered that variables from the criteria catalogue could also be appropriate as criteria for trauma room activation if they can be determined in a prehospital setting. In addition to the three criteria of the S3 guideline classified as Grade of Recommendation (GoR) A, namely advanced airway, GCS < 9 and systolic blood pressure < 90 mmHg, the following criteria are of extended importance (Table 5):

- Resuscitation
- Insertion of a chest tube
- Administration of catecholamine
- Drop in GCS ≥ 2 points
- SpO_2_ < 90%
- Hypothermia < 35 °C
- Shock index > 0.9
- Respiratory rate < 9 or > 29

**Table 5:** Criteria of extended importance which can be determined in a prehospital setting

Criteria of extended importance
Resuscitation
Insertion of a chest tube
Administration of catecholamine
Drop in GCS ≥ 2 points
SpO_2_ < 90%
Hypothermia < 35 °C
Shock index > 0.9
Respiratory rate < 9 or > 29

*GCS* glasgow coma score, *SpO*_*2*_ saturation of peripheral oxygen

### Limitations

This is a retrospective analysis based on registry data. Availability of data was > 95% for most criteria but unsatisfactory for temperature and respiratory rate. The selected approach is not a final validation of the criteria list. On account of the data available in TraumaRegister DGU^®^, the endpoint was mortality. An important aspect for the evaluation of triage quality would be emergency interventions that stabilise the patient and prevent mortality. Another important aspect is organ function, which trauma room treatment aims to stabilise. Further studies should evaluate whether some criteria can be excluded and whether certain criteria combinations could be relevant. In addition, only patients who were entered in the TraumaRegister DGU were available for the evaluation of the consensus-based catalogue of criteria. Patients with undertriage and did not receive trauma team treatment were not part of this cohort.

## Conclusion

The criteria catalogue identified 99.6% of all trauma patients who were admitted to the hospital through the trauma room and then died during their hospital stay.

On the basis of the assumption that patients who die in hospital belong to the group of patients that should have been admitted through the trauma room and should have received trauma care, the consensus-based criteria catalogue has proven itself suitable for the evaluation of triage quality. With regard to other aspects such as the stabilisation of vital functions and functional outcome, further studies are needed for the validation of the catalogue. Further studies are necessary to evaluate whether some criteria can be excluded and whether certain criteria combinations are relevant. In addition, this post hoc consensus-based criteria catalogue can already be used as an evaluation tool for new and existing criteria for activating trauma teams.

## Data Availability

The datasets during and/or analysed during the current study available from the corresponding author on reasonable request.

## Abbreviations

AIS: Abbreviated injury score
SD: Standard deviation
Sektion NIS: Committee on Emergency Medicine, Intensive Care and Trauma Management
DGU: German Trauma Society
ISS: Injury severity score
CDC: Centers for disease control and prevention
ACS: American College of Surgeon
CPR: Cardiopulmonary resuscitation
ICU: Intensive care unit
SpO2: Saturation of peripheral oxygen
GCS: Glasgow coma scale
D: Days
